# Stearoyl CoA desaturase is a gatekeeper that protects human beta cells against lipotoxicity and maintains their identity

**DOI:** 10.1007/s00125-019-05046-x

**Published:** 2019-12-03

**Authors:** Masaya Oshima, Séverine Pechberty, Lara Bellini, Sven O. Göpel, Mélanie Campana, Claude Rouch, Julien Dairou, Cristina Cosentino, Federica Fantuzzi, Sanna Toivonen, Piero Marchetti, Christophe Magnan, Miriam Cnop, Hervé Le Stunff, Raphaël Scharfmann

**Affiliations:** 1 Université Paris Descartes, Institut Cochin, Inserm U1016, 123 bd du Port-Royal, 75014 Paris, France; 2 Unité Biologie Fonctionnelle et Adaptative, CNRS UMR 8251, Paris, France; 3 Bioscience Metabolism, Research and Early Development Cardiovascular Renal and Metabolism, BioPharmaceuticals R&D, AstraZeneca, Gothenburg, Sweden; 4 Université Paris Descartes CNRS UMR 8601, Paris, France; 5 ULB Center for Diabetes Research, Université Libre de Bruxelles, Brussels, Belgium; 6 University of Pisa, Department of Clinical and Experimental Medicine, Pisa, Italy; 7 Division of Endocrinology, ULB Erasmus Hospital, Université Libre de Bruxelles, Brussels, Belgium; 8 Université Paris-Sud, CNRS UMR 9197, Institut des Neurosciences Paris-Saclay (Neuro-PSI) - CNRS UMR 9197, Orsay, France

**Keywords:** Dedifferentiation, Human, Lipotoxicity, Pancreatic beta cell, Type 2 diabetes

## Abstract

**Aims/hypothesis:**

During the onset of type 2 diabetes, excessive dietary intake of saturated NEFA and fructose lead to impaired insulin production and secretion by insulin-producing pancreatic beta cells. The majority of data on the deleterious effects of lipids on functional beta cell mass were obtained either in vivo in rodent models or in vitro using rodent islets and beta cell lines. Translating data from rodent to human beta cells remains challenging. Here, we used the human beta cell line EndoC-βH1 and analysed its sensitivity to a lipotoxic and glucolipotoxic (high palmitate with or without high glucose) insult, as a way to model human beta cells in a type 2 diabetes environment.

**Methods:**

EndoC-βH1 cells were exposed to palmitate after knockdown of genes related to saturated NEFA metabolism. We analysed whether and how palmitate induces apoptosis, stress and inflammation and modulates beta cell identity.

**Results:**

EndoC-βH1 cells were insensitive to the deleterious effects of saturated NEFA (palmitate and stearate) unless stearoyl CoA desaturase (SCD) was silenced. SCD was abundantly expressed in EndoC-βH1 cells, as well as in human islets and human induced pluripotent stem cell-derived beta cells. SCD silencing induced markers of inflammation and endoplasmic reticulum stress and also *IAPP* mRNA. Treatment with the SCD products oleate or palmitoleate reversed inflammation and endoplasmic reticulum stress. Upon SCD knockdown, palmitate induced expression of dedifferentiation markers such as *SOX9*, *MYC* and *HES1*. Interestingly, SCD knockdown by itself disrupted beta cell identity with a decrease in mature beta cell markers *INS*, *MAFA* and *SLC30A8* and decreased insulin content and glucose-stimulated insulin secretion.

**Conclusions/interpretation:**

The present study delineates an important role for SCD in the protection against lipotoxicity and in the maintenance of human beta cell identity.

**Data availability:**

Microarray data and all experimental details that support the findings of this study have been deposited in in the GEO database with the GSE130208 accession code.

**Electronic supplementary material:**

The online version of this article (10.1007/s00125-019-05046-x) contains peer-reviewed but unedited supplementary material, which is available to authorised users.

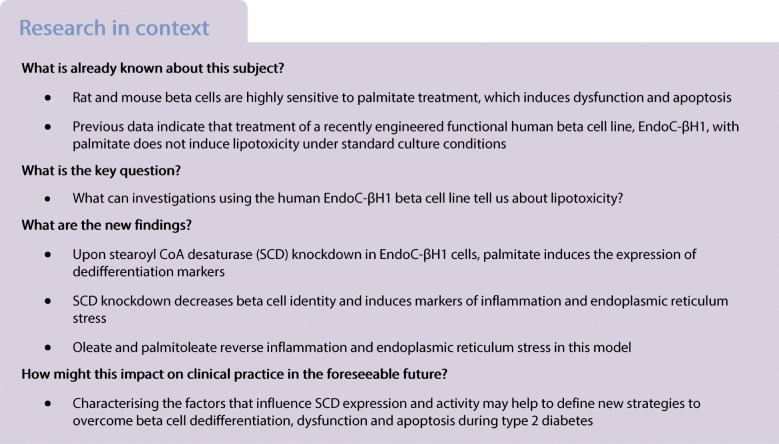



## Introduction

Type 2 diabetes develops as a consequence of a combination of insulin resistance of peripheral tissues and progressive decrease of functional pancreatic beta cell mass. This deficit is manifested by inadequate and insufficient insulin secretion in response to increased circulating glucose levels [1, 2]. Insulin resistance often precedes the development of type 2 diabetes, but it is now well established that pancreatic beta cell failure is a sine qua non condition for hyperglycaemia and type 2 diabetes to develop [1, 2].

NEFA represent an important source of energy for pancreatic beta cells in the normal state but can induce beta cell dysfunction and death when present in excessive levels during a prolonged period [1–3]. Chronic availability of fatty acids causes cell death and dysfunction in rodent beta cell lines [4, 5], isolated rodent islets and primary beta cells [6, 7], and animal models of diabetes [3, 8]. Several studies pointed out that the degree of NEFA saturation is important since saturated NEFA (e.g. palmitate or stearate) cause marked apoptosis, whereas unsaturated NEFA (e.g. palmitoleate or oleate) are much less cytotoxic and protect against saturated NEFA-mediated toxicity [7, 9–11]. The chronic adverse effects of saturated NEFA on beta cell function and viability are potentiated by the presence of hyperglycaemia, a phenomenon that is particularly seen in rodent beta cells and that has been termed ‘glucolipotoxicity’ [12, 13]. Numerous studies have suggested different mechanisms by which NEFA mediate beta cell dysfunction and death such as endoplasmic reticulum (ER) stress [14], increased intracellular triacylglycerol [15], reactive oxygen species (ROS) [16, 17], inflammation [14] and de novo synthesis of ceramide [15].

So far, the vast majority of data on the role of NEFA in beta cells has been derived from rodent models, either primary islets or rat and mouse beta cell lines [4, 18–20], with a more limited number of investigations performed using primary human islets [10, 14, 15, 21–26]. This is mainly due to the limited access to human islet preparations, which not only contain variable numbers of beta cells from one preparation to the other, but are also contaminated with non-endocrine cells such as exocrine tissue [27].

In this study, we sought to investigate lipotoxicity in a recently engineered functional human beta cell line, EndoC-βH1 [28]. This line represents a precious tool to study human beta cells in pathophysiological conditions [29]. As an example, EndoC-βH1 cells react to cytokine exposure in a similar manner to primary human beta cells [30]. Moreover, this cell line is suitable for drug screening [31].

## Methods

### Culture of EndoC-βH1 cells and treatment

EndoC-βH1 cells (Univercell Biosolutions, Toulouse, France [mycoplasma negative]) were cultured as described [28]. They were treated with 400 μmol/l of NEFA (palmitate, stearate, oleate and/or palmitoleate), in the presence of 5.6 mmol/l (low glucose) or 30 mmol/l glucose (high glucose [HG]), for the indicated periods (24 h to 72 h). NEFA was administered to the cells as a conjugate with fatty acid-free BSA. NEFA/BSA complex was prepared as described [12]. The molar ratio of NEFA to BSA was 5:1. The NEFA stock solutions were diluted in DMEM to obtain a 0.4 mmol/l final concentration at a fixed concentration of 0.5% BSA (low glucose and HG plus or minus NEFA). Unconjugated BSA was used as control. In some experiments, EndoC-βH1 cells were treated for 24 h with 500 μmol/l palmitate pre-complexed to NEFA-free BSA (Roche, Neuilly-sur-Seine, France) in medium supplemented with 1% FBS. EndoC-βH1 cells were treated with 5 μmol/l thapsigargin for 24 h (Sigma-Aldrich, Saint Quentin Fallavier, France). EndoC-βH1 cells were passaged and transfected using Lipofectamine RNAiMAX (Life Technologies, Saint Aubin, France) 24 h later as described [32, 33]. SMARTpool siRNAs for human *ELOVL6* (L-008861-01-0005), *SCD* (L-005061-00-0020), *SCD5* (L-008416-00-0005) or *SOX9* (M-021507-00-0020), or ON-TARGETplus non-targeting control pool siRNA (siCTRL, D-001810-01-20) were used (Dharmacon, GE Healthcare Life Sciences, Velizy-Villacoublay, France) at a final concentration of 80 nmol/l. In some experiments, EndoC-βH1 cells were transfected as described [33] with 30 nmol/l control siRNA (Qiagen, Antwerp, Belgium) or three different siRNAs targeting *SCD* (si*SCD*; electronic supplementary material [ESM] Table 1, ThermoFisher, Merelbeke, Belgium). *SCD* knocked down EndoC-βH1 cells will be hereafter referred to as βH1-SCD^KD^. *CPT1A*-targeting siRNA was purchased from ThermoFisher and was also used at a final concentration of 80 nmol/l (ThermoFisher, AM16708-10564). Briefly, siRNA and Lipofectamine RNAiMAX were combined in OptiMEM and applied to the cells. Medium was changed 2.5 h later for fresh EndoC-βH1 culture medium. Efficiency of gene knockdown was validated by qRT-PCR (quantitative real-time qPCR) and protein level (for stearoyl CoA desaturase [SCD] and SRY-box transcription factor 9 [SOX9]).

### Human islet culture

Pancreases were obtained with informed written consent and processed with the approval of the local ethics committee of the University of Pisa. Human islets were isolated at the University of Pisa, Italy, using collagenase digestion and density gradient purification from heart-beating organ donors [34]. The organ donors (three men, five women, age 67 ± 8 years [mean ± SD], BMI 27.3 ± 4.0 kg/m^2^, cause of death cerebral haemorrhage in six, stroke in one and post-anoxic encephalopathy in one) did not have a medical history of diabetes. Human islets were cultured in Ham’s F-10 medium as described [14]. Beta cell purity, evaluated by insulin immunocytochemistry in dispersed islet cells, was 47 ± 10%. Information on human islets is available in the Human Islets checklist in the ESM.

### Human induced pluripotent stem cell culture and differentiation into beta cells

The previously described human induced pluripotent stem cell (iPSC) line HEL115.6 [35] was differentiated into beta cells using a seven-stage protocol that makes use of monolayer culture on Matrigel-coated plates up to pancreatic progenitor stage 4 and then moves the cells to suspension culture until the last stage of beta cell differentiation [35]. Stage 7 aggregates contained 41 ± 14% beta cells (assessed by insulin immunocytochemistry).

### Assessment of cell death

Live/dead cells were counted following Trypan Blue staining. Caspase 3/7 activity assays were performed using the Promega Apo-ONE Homogenous caspase-3/7 Assay kit as described [36] (Promega, Charbonières-les-Bains, France). As another method for apoptosis detection, cells were stained with the Hoechst 33342 (5 μg/ml, Sigma-Aldrich) and propidium iodide (PI, 5 μg/ml, Sigma-Aldrich) and counted by fluorescence microscopy [37]. The xCELLigence system (ACEA Biosciences, San Diego, CA, USA), which is based on the continuous real-time monitoring of cell adhesion, was used for real-time and label-free monitoring of cell viability and growth [38]. Briefly, EndoC-βH1 cells were seeded into 96-well E-plates coated with extracellular matrix and fibronectin (50,000 cells/well), transfected with siRNA, treated with NEFA or BSA 72 h later and monitored for up to 72 h.

### Insulin content and glucose-stimulated insulin secretion

Insulin content and glucose-stimulated insulin secretion **(**GSIS) were measured as described [39].

### RNA isolation, reverse transcription, qRT-PCR and transcriptomic analyses

qRT-PCR was performed as described [32]. *ACTB* or *PPIA* transcript levels were used as housekeeping genes for normalisation. Primer sequences are listed in ESM Table 2. Global transcriptomic analyses were performed using the Affymetrix 2.0ST gene chip as described [32] (Affymetrix-Thermofisher, Courtaboeuf, France). Microarray data and all experimental details are available in the Gene Expression Omnibus (GEO) database (accession GSE130208). Heatmap analyses were generated using web-based Morpheus tool (https://software.broadinstitute.org/morpheus/; access date: 3 January 2019).

### Human IAPP promoter analysis

The 797 bp upstream sequence of the *IAPP* gene, which encodes islet amyloid polypeptide (IAPP), was extracted from NCBI Map viewer/Ace view, and scanned for the presence of SOX9 binding motifs using MatInspector (Genomatix software, https://www.genomatix.de/, access date: 3 January 2019; [40]). Results are presented in ESM Table 3.

### Measurement of NEFA levels by GC-MS

Cellular saturated and unsaturated NEFA levels were determined by GC-MS as described [41]. Briefly, cells were mixed with BF3 (14%)/methanol and heated (100°C; 40 min). Then, NEFA were extracted using heptane/distilled water (1∶2). NEFA present in the supernatant were evaporated and solubilised in heptane. NEFA methyl esters (1μl) were analysed on GC-MS instrument (Shimadzu interfaced with a GC2010 mass selective detector). Heptadecanoic acid was used as internal standard. The mass spectra and retention indices registered in the Fatty Acid Methyl Esters (FAMEs) GC/MS Library were obtained using the Shimadzu GCMS-QP2010 (Shimadzu, Marne-la-Vallée, France, https://www.shimadzu.fr, GCMSsolution Ver. 2) .

### Immunoblotting

Western blots were performed as described [32] using the following antibodies diluted in TBS 3% BSA 0.1% Tween-20 (Sigma-Aldrich): poly-(ADP-ribose) polymerase (PARP) (1/1000; 5625S; Cell Signaling, Saint-Cyr-L’École, France), SCD (1/500; M38; Cell Signaling), MafA (1/500; gift from A. Rezania, BetaLogics, Cambridge, MA, USA), SOX9 (1/500; ab5535; Millipore, Molsheim, France), DDIT3 (1/1000; 5554 Cell Signaling), tubulin (1/2000; T9026; Sigma-Aldrich) and actin (1/2000; A5441; Sigma-Aldrich). Antibodies were validated by knockdown experiments (SCD, SOX9, MAFA) or have passed application-specific testing standards (PARP, DDIT3, actin, tubulin). Species-specific HRP-linked secondary antibodies (1/1000; 7074 and 7076; Cell Signaling) were used.

### Statistical analyses

Graphs were constructed by using PRISM6 software (GraphPad, San Diego, CA, USA). Quantitative data are presented as the mean ± SD from three independent experiments. Results were analysed by one-way ANOVA with post hoc Tukey testing for multiple conditions or by *t* test if only two conditions were being tested (two-tailed). Randomisation and blinding were not carried out. A *p* value less than 0.05 was considered significant.

## Results

### EndoC-βH1 cells are resistant to palmitate toxicity

**Fig. 1:**
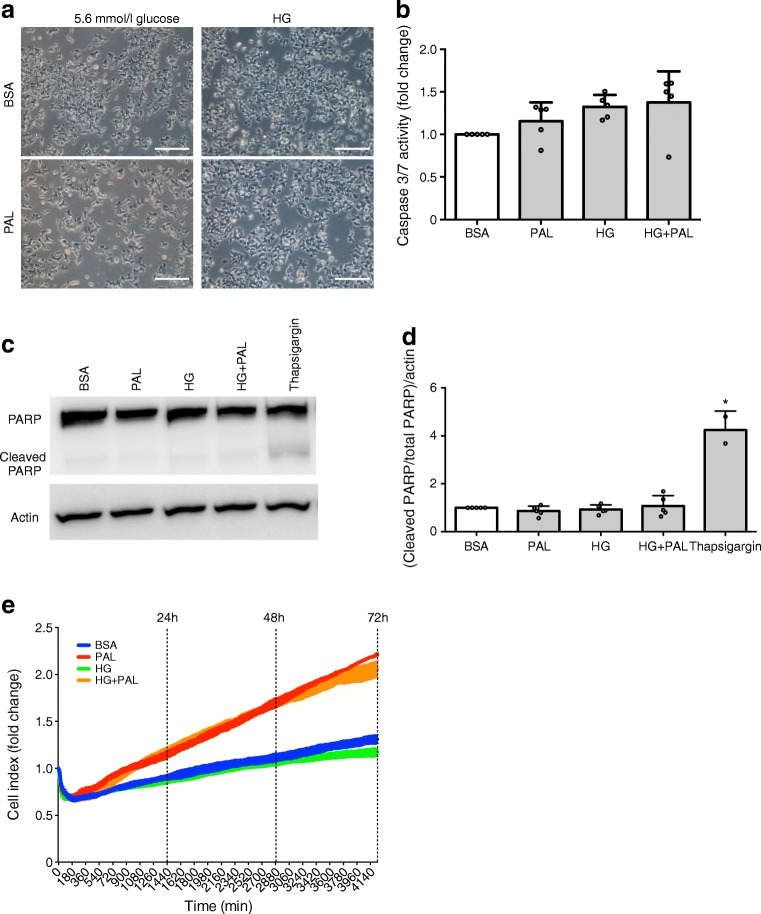
Palmitate and high glucose do not induce EndoC-βH1 cell death. EndoC-βH1 cells were either treated with BSA (control), 400 μmol/l palmitate (PAL), 30 mmol/l glucose (HG) or HG+PAL for 24 h. (**a**) Cell morphology. Representative images of cellular aspects after 24 h of treatment (representative images of three independent experiments; scale bars, 250 μm). (**b**) Apoptosis was measured by caspase3/7 cleavage activity (*n* = 5). (**c**, **d**) Representative western blot of PARP cleavage (**c**) and relative quantification of (cleaved PARP/total PARP) over actin (**d**) (*n* = 3–5). Thapsigargin treatment was used as positive control. (**e**) Real-time cell survival during a 72 h period after BSA, PAL, HG, or HG+PAL treatment measured by xCELLigence technology (representative of one experiment in triplicate). Data represent the means ± SD. *p < 0.05 relative to control (BSA)

We first analysed the effect of palmitate on EndoC-βH1 cell viability. We did not observe lipotoxicity associated with morphological changes or obvious cell death (characterised by floating cells or debris) in EndoC-βH1 cells treated with 0.4 mmol/l palmitate (C16:0). The concept of glucolipotoxicity, i.e. the deleterious effects of combined elevated glucose and NEFA concentrations, prompted us to study EndoC-βH1 cell viability following both high glucose and NEFA exposure. The efficiency of HG (30 mmol/l) treatment was validated by *TXNIP* mRNA upregulation ([39] and data not shown). Remarkably, we did not observe cell toxicity after palmitate incubation at low glucose (5.6 mmol/l) or HG (Fig. 1a). To strengthen our investigation, we measured caspase 3/7 cleavage as another marker of cells undergoing apoptosis. Accordingly, we did not observe changes in caspase 3/7 cleavage activity upon palmitate exposure (Fig. 1b). We then quantified PARP cleavage, another apoptosis-related measurement. Thapsigargin induced cell apoptosis as determined by increased PARP cleavage, but this was not the case with palmitate (Fig. 1c, d). Finally, to survey the effects of palmitate over a prolonged period of time (up to 72 h) in real time, we used the xCELLigence system. Palmitate treatment did not decrease cell proliferation/survival, but, in fact, it increased it in a time-dependent manner (Fig. 1e).

These data indicate that long chain saturated NEFA such as palmitate, with or without HG, do not induce glucolipotoxicity in EndoC-βH1 cells.

### SCD is involved in EndoC-βH1 protection against lipotoxicity

**Fig. 2:**
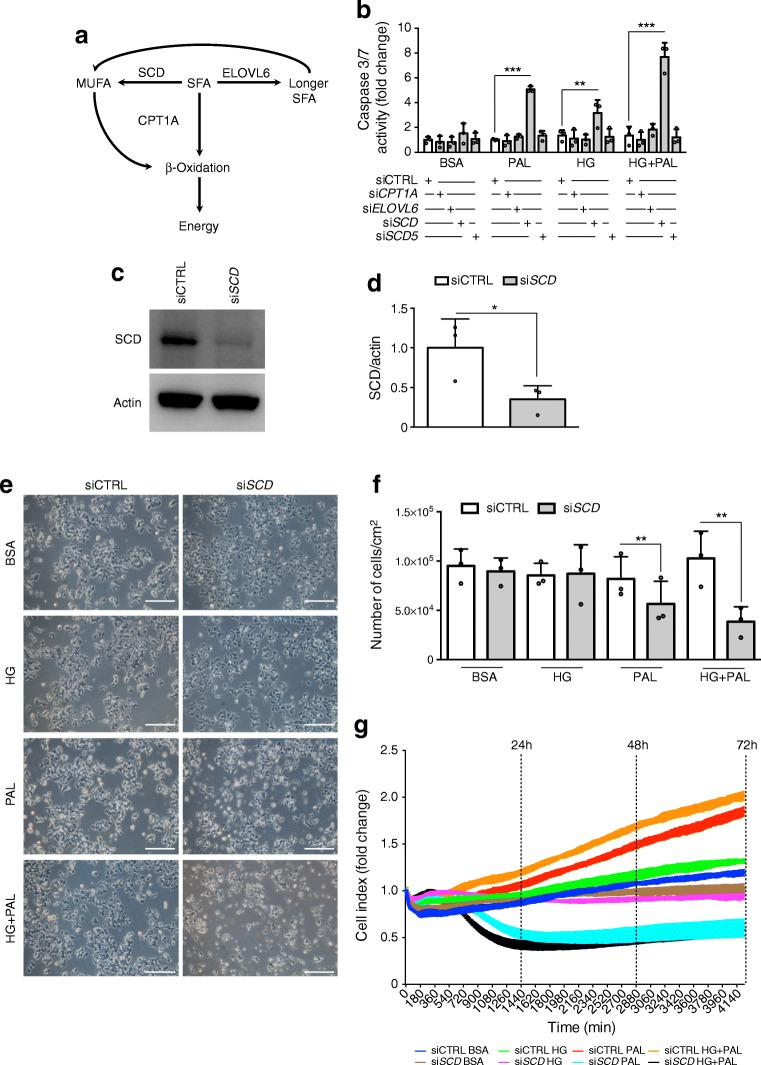
Palmitate and high glucose induce βH1-SCD^KD^ cell death. (**a**) Schematic representation of enzymes involved in palmitate metabolism. CPT1A, carnitine palmitoyltransferase 1A; MUFA, mono-unsaturated fatty acid; SFA, saturated fatty acid. (**b**) EndoC-βH1 cells were transfected with siCTRL, *CPT1A*-targeting siRNA (si*CPT1A*), *ELOVL6*-targeting siRNA (si*ELOVL6*), *SCD*-targeting siRNA (si*SCD*) or *SCD5*-targeting siRNA (si*SCD5*) for 72 h and then treated with BSA (control), 400 μmol/l palmitate (PAL), 30 mmol/l glucose (HG) or HG+PAL for 24 h. Apoptosis was measured by caspase 3/7 cleavage activity (*n* = 3). (**c**, **d**) SCD immunoblotting and quantification following si*SCD* treatment in EndoC-βH1 cells (representative western blot of three independent experiments). (**e**–**g**) EndoC-βH1 cells were transfected with siCTRL or si*SCD* and treated 72 h later with BSA, HG, PAL or HG+PAL for 24–72 h. (**e**) Images represent cellular morphology 24 h after treatment (representative images of three independent experiments; scale bars, 250 μm). (**f**) Cell counts after treatment (*n* = 3). (**g**) Real-time cell survival during a 72 h period after BSA, HG, PAL or HG+PAL treatment measured by xCELLigence technology (representative of two experiments in triplicate). Data represent the means ± SD. *p < 0.05, **p < 0.01 and ***p < 0.001 relative to control as indicated on the graph

Real-time monitoring using xCELLigence suggested that palmitate may in fact increase cell proliferation/survival (Fig. 1). Palmitate can either enter the mitochondrial NEFA β-oxidation pathway, or be elongated and then desaturated to be incorporated into neutral lipids, two pathways known to be protective to cells (Fig. 2a, [13, 14]). We tested whether altering the enzymes involved in palmitate metabolism modifies the effects of NEFA on EndoC-βH1 cells. We performed knockdown using siRNA against: *CPT1A*, the rate-limiting-step enzyme of NEFA β-oxidation; *ELOVL6*, which elongates palmitate into stearate; and *SCD* and *SCD5*, which desaturate palmitate or stearate into palmitoleate (C16:1) or oleate (C18:1), respectively. Each siRNA was specific and efficient (>50% downregulation in the mRNA target) (ESM Fig. 1a). siRNA-transfected EndoC-βH1 cells were next treated with palmitate ± HG. Upon *CPT1A* and *ELOVL6* knockdown, palmitate did not induce caspase 3/7 cleavage (Fig. 2b). But upon *SCD* knockdown (Fig. 2c,d, ESM Fig. 1a), palmitate treatment increased caspase 3/7 cleavage in EndoC-βH1 cells (Fig. 2b). To rule out off-target effects, we used three other siRNAs targeting different regions of the SCD mRNA (ESM Table 1, ESM Fig. 1b), and these consistently sensitised EndoC-βH1 cells to palmitate-induced apoptosis measured by Hoechst 33342 and PI staining (ESM Fig. 1c). Of note, upon *SCD5* knockdown, another SCD isoform expressed by human beta cells, palmitate ± HG did not induce toxicity (Fig. 2b). Moreover, palmitate ± HG treatment of βH1-SCD^KD^ cells decreased cell survival as measured by cell morphology, cell counts and xCELLigence (Fig. 2e, g). Similar results were obtained with stearate (C18:0), another long chain saturated NEFA (ESM Fig. 2a, b). Of note, real-time qPCR quantification indicated that, in EndoC-βH1 cells, *SCD* mRNA expression was high (C_t_ ~19) when compared with other enzymes implicated in saturated NEFA metabolism (*CPT1A*: C_t_ ~26; *ELOVL6*: C_t_ ~24; *SCD5*: C_t_ ~25). Its expression was also high in human islets and in iPSC-derived beta cells, with an increase in the last stage of human beta cell maturation in this in vitro model of pancreatic endocrine cell development (ESM Fig. 3).

Thus, SCD, an enzyme that catalyses a rate-limiting step in the synthesis of unsaturated NEFA, is involved in EndoC-βH1 cell protection against (gluco)lipotoxicity induced by palmitate and stearate.

### Long chain saturated NEFA modulate the expression of stress-related genes in βH1-SCD^KD^ cells

**Fig. 3:**
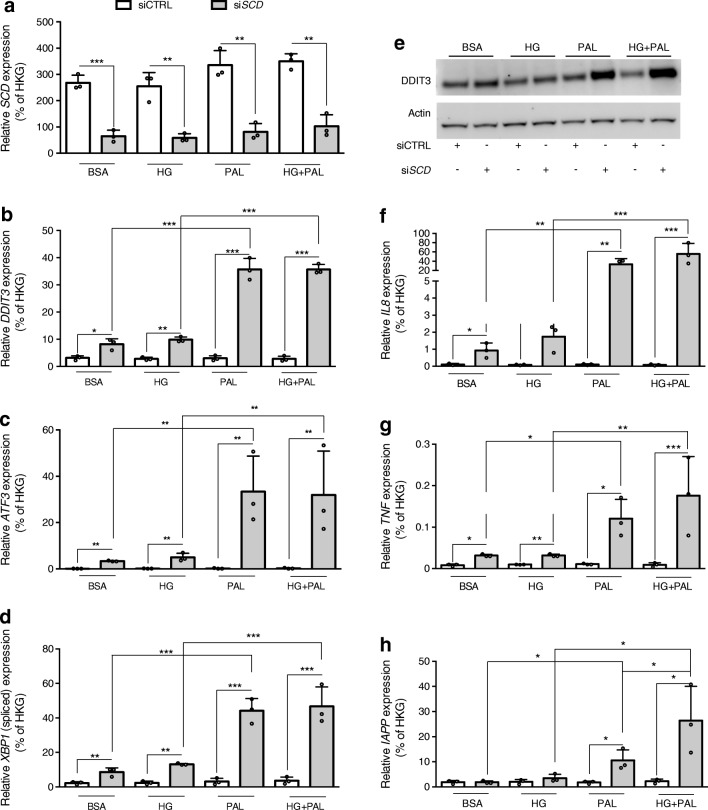
Palmitate modulates gene expression in βH1-SCD^KD^ cells. EndoC-βH1 cells were transfected with siCTRL or si*SCD* and treated 72 h later with BSA (control), 400 μmol/l palmitate (PAL), 30 mmol/l glucose (HG) or HG+PAL for 24 h. qRT-PCR data show mRNA expression of (**a**) *SCD*, and (**b**–**d**) the ER stress-related genes *DDIT3* (**b**), *ATF3* (**c**) and the spliced variant of *XBP1* (**d**). (**e**) Western blot of DDIT3 (representative western blot of three independent experiments). (**f**–**h**) qRT-PCR data show mRNA expression of the proinflammatory genes *IL8* (**f**), *TNF* (**g**) and *IAPP* (**h**). mRNA expression is relative to housekeeping genes (HKG). The key in (**a**) is also applicable to (**b**–**d**) and (**f**–**h**). Data represent the means ± SD of three independent experiments. *p < 0.05, **p < 0.01 and ***p < 0.001 relative to control as indicated on the graph

We next analysed in βH1-SCD^KD^ cells the effects of palmitate (± HG) treatments on the expression of genes previously found to be upregulated by palmitate in human islets, such as genes related to ER stress (*ATF3*, *DDIT3*, spliced variant of *XBP1*) and inflammation (*IL8*, *TNF*) and also *IAPP* [14]. When EndoC-βH1 cells were transfected with a control siRNA, we did not observe upregulation of the aforementioned genes upon palmitate treatment (± HG), confirming the lack of lipotoxicity. However, palmitate treatment of βH1-SCD^KD^ cells induced *ATF3*, *DDIT3*, spliced *XBP1*, *IL8*, *TNF* and *IAPP* mRNAs (Fig. 3a–d, f–h). Similar inductions were observed with stearate (C18:0) treatment (ESM Fig. 4). ER stress marker DDIT3 was also induced at the protein level (Fig. 3e). Of note, ER stress- and inflammation-related gene expression was already induced upon *SCD* knockdown by itself, suggesting that the inhibition of endogenous NEFA desaturation is sufficient to elicit mild ER and inflammatory stress; exogenous palmitate or stearate treatment further enhanced these inductions (Fig. 3b–d, f–g, ESM Fig. 4) [23].

These data indicate that following *SCD* knockdown, EndoC-βH1 cells respond to palmitate and stearate in a way similar to that observed in human islets.

### Palmitate-induced *IAPP* upregulation in βH1-SCD^KD^ cells requires SOX9

**Fig. 4:**
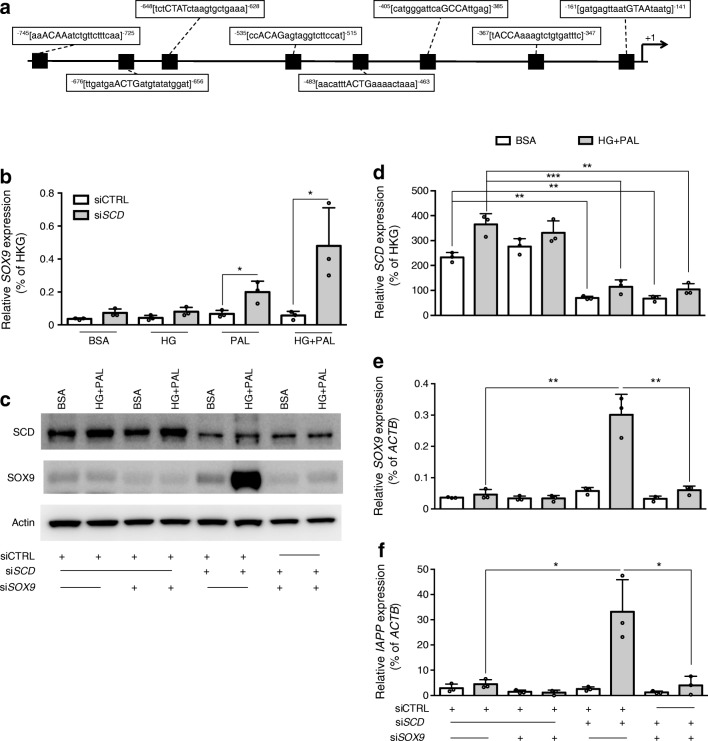
Palmitate-induced IAPP expression in βH1-SCD^KD^ cells is dependent on SOX9. (**a**) Schematic representation of the *IAPP* promoter showing potential SOX9 binding sites identified with MatInspector (Genomatix software). The numbers refer to the nucleotide position upstream of the transcription start site (+1). SOX9 binding motifs are shown in upper case letters. (**b**) EndoC-βH1 cells were transfected with either siCTRL or si*SCD* and treated 72 h later with BSA (control), 400 μmol/l palmitate (PAL), 30 mmol/l glucose (HG) or HG+PAL for 24 h. qRT-PCR data show *SOX9* mRNA expression relative to housekeeping genes (HKG). (**c**–**f**) EndoC-βH1 cells were either transfected with siCTRL, si*SOX9*, si*SCD* or si*SCD*+si*SOX9*. Seventy-two hours later, they were treated with BSA or HG+PAL for 24 h. (**c**) western blot analysis of SCD and SOX9 expression (representative western blot of three independent experiments). (**d**–**f**) qRT-PCR data show mRNA expression (relative to *ACTB*) of *SCD* (**d**), *SOX9* (**e**) and *IAPP* (**f**). The *x*-axis conditions below (**f**) also apply to (**d**, **e**) and the key above (**d**) also applies to (**e**, **f**). Data represent the means ± SD of three independent experiments. *p < 0.05, **p < 0.01 and ***p < 0.001 relative to control as indicated on the graph

*IAPP* is upregulated in several dysfunctional beta cell models. Genomatix analysis suggested eight potent SOX9 binding sites in the human *IAPP* promoter (Fig. 4a and ESM Table 3). SOX9 is a transcription factor expressed in pancreatic progenitors and in duct cells in the adult pancreas but also in beta cells upon dedifferentiation [32, 42–44]. Here, we observed that *SOX9* expression was significantly upregulated in palmitate-treated βH1-SCD^KD^ cells at the mRNA and protein levels (Fig. 4b, c). To study SOX9 involvement in *IAPP* induction, we prevented SOX9 induction using siRNA in βH1-SCD^KD^ cells (Fig. 4c–e) and then treated these cells with palmitate + HG. Under this setting, *IAPP* induction by palmitate + HG was abolished (Fig. 4f).

Our data thus demonstrate that upregulation of *IAPP* by palmitate + HG requires the induction of the beta cell dedifferentiation marker SOX9.

### Dedifferentiation is observed upon SCD knockdown

**Fig. 5:**
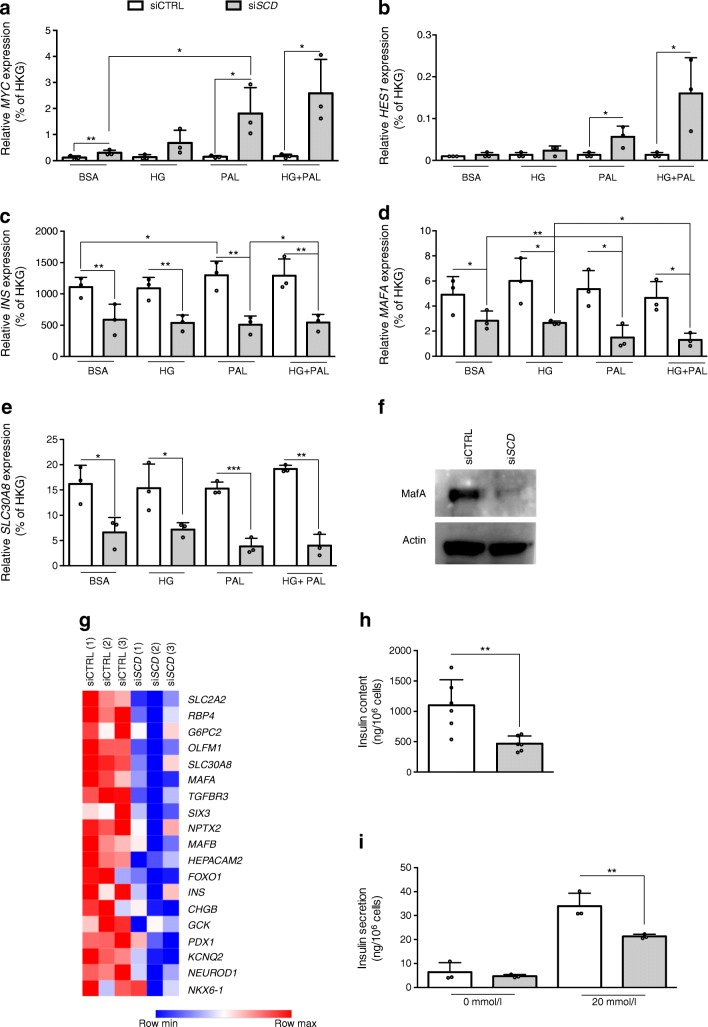
Palmitate exacerbates dedifferentiation of βH1-SCD^KD^ cells. EndoC-βH1 cells were transfected with siCTRL or si*SCD* and treated 72 h later with BSA, 400 μmol/l palmitate (PAL), 30 mmol/l glucose (HG) or HG+PAL for 24 h. (**a**–**b**) qRT-PCR data show mRNA expression of the dedifferentiation markers *MYC* (**a**) and *HES1* (**b**) (*n* = 3). (**c**–**e**) qRT-PCR data show mRNA expression of the beta cell markers *INS* (**c**), *MAFA* (**d**) and *SLC30A8* (**e**) (*n* = 3). (**f**) Western blot analysis of MafA expression (representative western blot of three independent experiments). (**g**) Heatmap of beta cell genes upon *SCD* downregulation (three separate samples for each siRNA). (**h**, **i**) Effects of *SCD* knockdown on GSIS in EndoC-βH1 cells. EndoC-βH1 cells were transfected with siCTRL or si*SCD*. Insulin content (**h**; *n* = 6) and secretion (**i**; *n* = 3) were assessed 6 days later by stimulation of EndoC-βH1 cells with 0 mmol/l or 20 mmol/l glucose. The key in (**a**) also applies to (**b**–**e**) and (**h**, **i**). Data represent the means ± SD. *p < 0.05, **p < 0.01 and ***p < 0.001 relative to control as indicated on the graph

We next investigated other described beta cell dedifferentiation markers [32, 42]. We observed *HES1* and *MYC* upregulation in palmitate-treated βH1-SCD^KD^ cells (Fig. 5a, b). At the same time, the expression of the beta cell-specific markers *INS*, *MAFA* and *SLC30A8* sharply decreased (Fig. 5c–e). Surprisingly, their expression was already downregulated in βH1-SCD^KD^ cells alone (without palmitate treatment) (Fig. 5c–f), suggesting that *SCD* depletion is sufficient to induce EndoC-βH1 cell dedifferentiation. RNA microarray analysis indicated the downregulation of additional beta cell markers such as *G6PC2*, *SLC2A2* and *FOXO1* in βH1-SCD^KD^ cells (Fig. 5g), further supporting beta cell dedifferentiation [32, 42, 45]. We did not observe any upregulation of non-beta cell endocrine cell markers such as *GCG* or *SST* or exocrine markers such as *HNF1B* and *PTF1A* (ESM Fig. 5)*.* Finally, insulin content decreased following SCD downregulation (Fig. 5h). Moreover, GSIS was reduced by 38% in βH1-SCD^KD^ cells (Fig. 5i).

### Induction of inflammation and ER stress in βH1-SCD^KD^ cells is reduced by oleate and palmitoleate treatment

SCD is the rate-limiting enzyme that catalyses the production of palmitoleate/oleate from palmitate/stearate. MS analysis indicated that SCD knockdown in EndoC-βH1 cells decreased basal oleate concentrations with a significant decrease in the oleate/stearate ratio (Table 1). Of note, we did not observe a decrease in basal palmitoleate concentrations after SCD knockdown compared with siCTRL (Table 1), suggesting that SCD is primarily transforming stearate into oleate in EndoC-βH1 cells. Moreover, elongation of C16 into C18 NEFA by ELOVL6 might be an important step for long chain fatty acid metabolism in EndoC-βH1 cells. Remarkably, *ELOVL6* is slightly upregulated upon SCD knockdown (ESM Fig. 1). However, co-transfection of *SCD* and *ELOVL6* siRNAs did not reverse dedifferentiation, inflammation and ER stress, suggesting that the degree of NEFA saturation is more important than length in conferring toxicity (data not shown).

**Table 1:** Lipid content in whole EndoC-βH1 cell lysates following siCTRL or si*SCD* transfection

Fatty acids (μg/10^6^ cells)	EndoCβH1-siCTRL	βH1-SCD^KD^
Palmitate	3.084 ± 0.156	2.788 ± 0.110
Palmitoleate	2.088 ± 0.184	2.236 ± 0.146
Stearate	1.351 ± 0.069	1.522 ± 0.036
Oleate	5.766 ± 0.209	3.703 ± 0.006***
Linoleate	0.411 ± 0.018	0.331 ± 0.001*
α-Linolenate	0.106 ± 0.041	0.168 ± 0.022
Arachidonic acid	0.095 ± 0.002	0.189 ± 0.027*
Eicosapentaenoic acid	0.076 ± 0.053	0.124 ± 0.008
Docosapentaenoic	0.027 ± 0.002	0.090 ± 0.044
Docosahexaenoic acid	3.682 ± 0.179	3.500 ± 0.528
Palmitoleate/palmitate	0.675 ± 0.030	0.801 ± 0.029
Oleate/stearate	4.275 ± 0.089	2.436 ± 0.055***

Data are means ± SD

*p < 0.05 and ***p < 0.001 relative to siCTRL

**Fig. 6:**
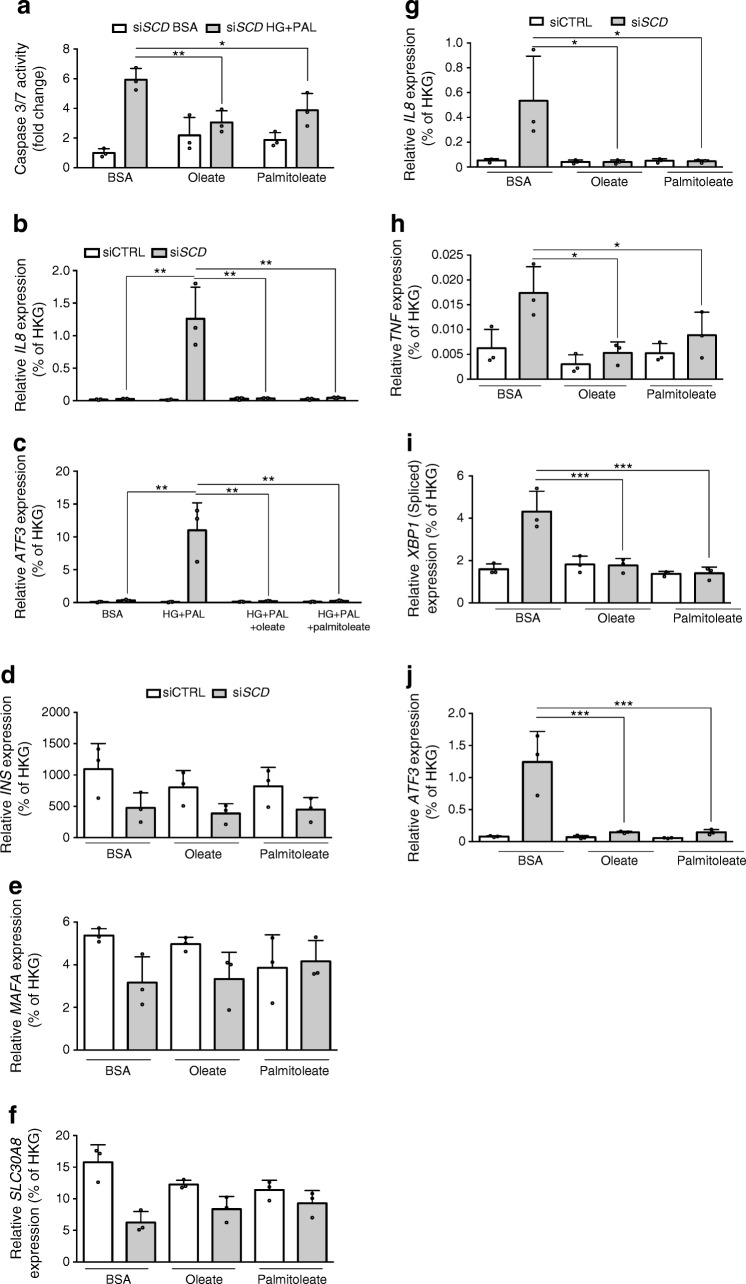
Monounsaturated fatty acids reduce gene expression induced in βH1-SCD^KD^ cells. (**a**) EndoC-βH1 cells were transfected with si*SCD* for 72 h, then treated with BSA or HG+PAL (30 mmol/l glucose, 400 μmol/l palmitate), and further co-treated with BSA, 400 μmol/l oleate or 400 μmol/l palmitoleate for 24 h. Apoptosis was measured by caspase 3/7 cleavage activity. (**b**–**c**) EndoC-βH1 cells were transfected with either siCTRL or si*SCD* for 72 h, then treated with BSA, HG+PAL, or HG+PAL with either oleate or palmitoleate (both 400 μmol/l) for 24 h; mRNA levels of *IL8* (**b**) and *ATF3* (**c**) were measured by qRT-PCR. The *x*-axis conditions below (**c**) also apply to (**b**). (**d**–**j**) EndoC-βH1 cells were transfected with either siCTRL or si*SCD* for 72 h, then treated with BSA, 400 μmol/l oleate or 400 μmol/l palmitoleate for 24 h. qRT-PCR data show mRNA expression of the beta cell markers *INS* (**d**), *MAFA* (**e**) and *SLC30A8* (**f**), the inflammatory genes *IL8* (**g**) and *TNF* (**h**), and the ER stress genes *XBP1* (spliced variant) (**i**) and *ATF3* (**j**). The keys above (**d**) and (**g**) apply to all graphs below them. mRNA expression is relative to housekeeping genes (HKG). Data represent the means ± SD of three independent experiments. *p < 0.05, ** *p* < 0.01 and ***p < 0.001 relative to control as indicated on the graph

We next asked whether oleate or palmitoleate supplementation could reverse some phenotypic traits observed in βH1-SCD^KD^ cells*.* Treatment of βH1-SCD^KD^ cells with oleate and palmitoleate reduced the effects of palmitate/HG on caspase 3/7 cleavage activity that was paralleled by an absence of induction of *IL8* and *ATF3* (Fig. 6a–c). Finally, in the absence of palmitate/HG, while oleate and palmitatoleate did not reverse the *INS*, *MAFA* or *SLC30A8* downregulation observed upon SCD knockdown (Fig. 6d–f), the induction of inflammation (*IL8*, *TNF*) and ER stress (spliced *XBP1*, *ATF3*) markers was reduced (Fig. 6g–j).

## Discussion

Chronically elevated saturated NEFA levels can impair the function of pancreatic beta cells. The mechanisms involved in beta cell lipotoxicity induced by saturated NEFA are the subject of active investigations because of its association with the development of type 2 diabetes [2, 3]. However, our knowledge of how saturated NEFA act on human beta cells and induce diabetes is limited. Defining these mechanisms could help to develop new strategies to prevent beta cell dysfunction and death in type 2 diabetes. Rodent models have been useful to better understand the mechanisms of NEFA-induced beta cell dysfunction. However, differences exist between human and rodent beta cells in response to NEFA [21, 46, 47]. For example, palmitate differentially affects protein acetylation in rodent and human beta cells [47]. Remarkably, human islets appear to be more resistant to apoptosis than rodent RIN1046-38, INS-1 or Min6 cell lines [21, 46, 48, 49]. It is thus of major importance to develop human beta cell models of lipotoxicity. As access to primary human islet preparations is limited and variability exists from one human islet preparation to the other [27], we recently developed functional human beta cell lines [28, 50] and tested here their use in modelling human beta cell lipotoxicity.

Rat and mouse beta cells are highly sensitive to palmitate treatment that induces dysfunction and apoptosis [3]. On the other hand, previous data indicated that treatment of EndoC-βH1 cells with palmitate does not induce lipotoxicity under standard culture conditions [51, 52]. Our current data further confirm this. By investigating saturated NEFA metabolism and its related enzymes through knockdown using siRNA, we identified SCD as the main brake on palmitate toxicity. SCD is highly expressed in primary human beta cells ([50, 51] and the present study). Interestingly, elevated SCD levels have been shown to protect against saturated NEFA in a number of cell types, including the mouse beta cell line MIN6 cells and human islets [21, 48, 49]. The working hypothesis is that SCD rapidly desaturates palmitate/stearate into palmitoleate/oleate, and thus decreases their toxicity. Five different SCDs (SCD1–5) have been described in the mouse while there are only two in humans (SCD and SCD5) [53]. It is noteworthy that SCD5 is predominantly expressed in the human brain and pancreatic islets (beta and delta cells), human beta cell lines and pancreatic ductal cells ([53, 54] and the present study). Even though SCD5 has been shown to desaturate NEFA [55], our data indicate that, while SCD knockdown induces lipotoxicity in EndoC-βH1 cells upon palmitate treatment, this is not the case upon SCD5 knockdown. This suggests that, in human beta cells, SCD plays the dominant role in the desaturation of long chain saturated NEFA. Another possibility is that products of SCD and SCD5 are used for differential lipogenic reactions. Indeed, SCD is known to play a central role in the synthesis of neutral lipids such as triacylglycerol, which are protective for beta cells [11]. In contrast, in neuronal cells overexpressing SCD5, triacylglycerol and phosphatidylethanolamine formation was reduced whereas de novo synthesis of phosphatidylcholine and cholesteryl esters was increased [55]. Additional analyses are needed to unravel SCD5 function in human beta cells. Interestingly, SCD5 is involved in neuronal cell proliferation and differentiation [55] and in survival of MCF-7 cells, in which cancer-associated fibroblasts induced the expression of SCD5 [56].

Our study further shows that palmitate treatment of βH1-SCD^KD^ cells induced the expression of genes related to inflammation (*IL8*, *TNF)* and ER stress (*ATF3*, *DDIT3*, spliced *XBP1*). Increased phospholipid saturation upon inhibition of SCD could contribute to enhance ER stress in the presence of palmitate, as observed in HeLa cells [57]. These saturated lipids reduce ER membrane fluidity, which may secondarily lead to ER Ca^2+^ depletion, reduced protein folding and ER stress [37]. Palmitate also induced the expression of *IAPP* mRNA levels in βH1-SCD^KD^ cells, as previously observed in human islets treated with palmitate [14]. Remarkably, we found that the expression of SOX9, a beta cell dedifferentiation marker [32, 42, 44], was induced by palmitate in βH1-SCD^KD^ cells, as were *HES1* and *MYC*. SOX9 activation was necessary for the induction of *IAPP* by palmitate. Of note, amyloid deposits were recently described surrounding dedifferentiated beta cells in individuals with type 2 diabetes [58]. We propose that beta cell dedifferentiation and induction of SOX9 expression represents an early step that enhances IAPP expression. Human IAPP is co-expressed and co-secreted with insulin. In type 2 diabetes patients, IAPP forms cytotoxic ‘amyloid’ plaques within islets [59, 60]. This phenomenon is difficult to study in mice as rodent IAPP does not form amyloid fibres [59, 60]. Palmitate-treated βH1-SCD^KD^ cells may thus represent a new model to understand the regulation of *IAPP* expression and its potential to form deleterious amyloid fibres [60].

We observed that SCD knockdown by itself was sufficient to give rise to major phenotypes. It decreased the expression of central beta cell markers such as *INS*, *MAFA* and *SLC30A8*. These observations underline a new role for SCD in maintaining mature beta cell identity. It is noteworthy that SCD is also upregulated during beta cell maturation suggesting an important role in adult beta cell function and identity ([61, 62] and the present study). SCD knockdown reduced GSIS in EndoC-βH1 cells. Interestingly, it has been shown that extraction of NEFA with NEFA-free BSA from the plasma membrane of MIN6 cells reduced insulin secretion [63]. There, oleate was one of the most extracted NEFA, suggesting that its endogenous synthesis through SCD plays a central role in the regulation of insulin secretion in beta cells. SCD knockdown also induced markers of inflammation and ER stress in EndoC-βH1 cells. The beneficial effects of oleate compared with palmitic acid on insulin resistance and type 2 diabetes is well established [64]. In the present study, SCD knockdown decreased the ratio oleate/palmitate by 30%, suggesting that this reduction could contribute to the deleterious effect of palmitate in βH1-SCD^KD^ cells. In keeping with this, the induction of inflammatory (*IL8*, *TNF*) and ER stress (spliced *XBP1*, *ATF3*) markers was rescued upon addition of oleate and palmitoleate, the products of SCD enzyme reactions. On the other hand, treatment with oleate and palmitoleate did not rescue the expression of beta cell differentiation markers. Future experiments will test whether other conditions of treatment with oleate or palmitoleate (different concentrations, longer exposure time) will reverse the dedifferentiation phenotype observed upon SCD knockdown. Taken together, we propose that SCD is a gatekeeper in human beta cells that protects against dedifferentiation, dysfunction, inflammation and ER stress. βH1-SCD^KD^ cells thus represent an innovative model to discover pathways and molecules that maintain high levels of SCD and protect against such deleterious effects.

Many observations suggest that SCD is important for beta cell adaptation and compensation during type 2 diabetes development in rodents. *Scd1* and *Scd2* mRNA expression is induced in islets from prediabetic hyperinsulinaemic Zucker Diabetic Fatty rats and their expression decreases when diabetes develops [49]. Consistent with this observation, diet-induced obesity reduces *Scd1* mRNA expression in rodent islets [65]. Moreover, while global knockout of *Scd1* in mice improves insulin sensitivity, when introduced on the *ob/ob* background with leptin-deficiency, *Scd1* deletion leads to a worsening of diabetes [66]. Importantly, *SCD* gene expression was lower in beta cell enriched tissue (obtained by laser capture microdissection) from individuals with type 2 diabetes compared with healthy donors [67]. We propose that, over time, in the course of type 2 diabetes progression, SCD expression by beta cells is first induced during compensation in response to insulin resistance, and as the duration of diabetes increases, SCD expression decreases leading to a decline in beta cell function. Characterising the factors that influence SCD expression or activity, such as liver X receptor (LXR)/peroxisome proliferator-activated receptor α (PPARα), sterol regulatory element-binding protein 1c **(**SREBP-1c) and/or cholesterol [68, 69], will help us define new strategies to overcome beta cell dedifferentiation, dysfunction and death in type 2 diabetes. Our results described above will enable progress on this important topic using βH1-SCD^KD^ as a human beta cell model.

## Electronic supplementary material

ESM (PDF 3007 kb)

ESM Table 3 (XLS 149 kb)

## Data Availability

Microarray data and all experimental details that support the findings of this study have been deposited in in the GEO database with the GSE130208 accession code.

## Abbreviations

βH1-SCD^KD^: SCD knocked-down EndoC-βH1 (cells)
ER: Endoplasmic reticulum
GSIS: Glucose-stimulated insulin secretion
HG: High glucose
IAPP: Islet amyloid polypeptide
iPSC: Induced pluripotent stem cell
PARP: Poly-(ADP-ribose) polymerase
PI: Propidium iodide
qRT-PCR: quantitative real-time PCR
SCD: Stearoyl CoA desaturase
siCTRL: Control siRNA
SOX9: SRY-box transcription factor 9
