# Supplementary material for: Stearoyl CoA desaturase is a gatekeeper that protects human beta cells against lipotoxicity and maintains their identity
Source: Diabetologia. 2019 Dec 3;63(2):395–409. doi: 10.1007/s00125-019-05046-x (PMC6946759; doi:10.1007/s00125-019-05046-x)
Supplement: Supplementary file 1 — (PDF 3007 kb) [file 125_2019_5046_MOESM1_ESM.pdf]

ESM Table 1 siRNA table

siSCD#1: 5'-CCACCUCUUCGGAUAUCGUCCUUAU-3'
3'-GGUGGAGAAGCCUAUAGCAGGAAUA-5'
siSCD#2: 5'-CAGGACGAUAUCUCUAGCUCCUAUA-3'
3'-GUCCUGCUAUAGAGAUCGAGGAUAU-5'
siSCD#3: 5'-CAGGGUCCUGCAGAAUGGAGGAGAU-3'
3'-GUCCCAGGACGUCUUACCUCCUCUA-5'

ESM Table 2 Primers table

	**Fw**	**Rv**
ACTB	CTGTACGCCAACACAGTGCT	GCTCAGGAGGAGCAATGATC
ATF3	CTGCAAAGTGCCGAAACAAG	GCCTTCAGTTCAGCATTCACA
CMYC	GTAGTGGAAAACCAGCAGCC	AGAAATACGGCTGCACCGAG
CPT1A	CAGGCCGAAAACCCATGTTG	AGGCCTCACCGACTGTAGAT
DDIT3	GCTACTGACTACCCTCTCACTA	TACAAGCTGAGACCTTTCCTTT
ELOVL6	CTCAGCTACCTTGTGCTCTTC	CCTCCTCAGTTCCAACACTATTC
HES1	AGAAAGATAGCTCGCGGCATT	TACTTCCCCAGCACACTTGG
IAPP	TTGGTGCCATTCTCTCATCTAC	CAAGTAATTCAGTGGCTCTCTCT
IL8	AAATCTGGCAACCCTAGTCTG	GTGAGGTAAGATGGTGGCTAAT
INS	TGTCCTTCTGCCATGGCCCT	TTCACAAAGGCTGCGGCTGG
MAFA	ATTCTGGAGAGCGAGAAGTGCCAA	CGCCAGCTTCTCGTATTTCTCCTT
PPIA	ATGGCAAATGCTGGACCCAACA	ACATGCTTGCCATCCAACCACT
SCD (Pair 1)	ACAACTACCACCACTCCTTTC	GGAGACTTTCTTCCGGTCATAG
SCD (Pair 2)	TCCTATACCACCACCACCAC	GGGCCTTCCTTATCCTTGTA
SCD5	GGTGGCTGTTTGTTCGCAAG	ACCACAAAGCACATGAGCAC
SLC30A8	ACAGCCAAGTGGTTCGGAGAGAAA	TTGGGAAACTGACGGTGTGACTGA
SOX9	TTCACCTACATGAACCCCGC	AAGGTCGAGTGAGCTGTGTG
TNF	GATCCCTGACATCTGGAATCTG	GAAACATCTGGAGAGAGGAAGG
XBP1 (Spliced)	GCTGAGTCCGCAGCAGG	TGTCCAGAATGCCCAACAGG

ESM Figure 1

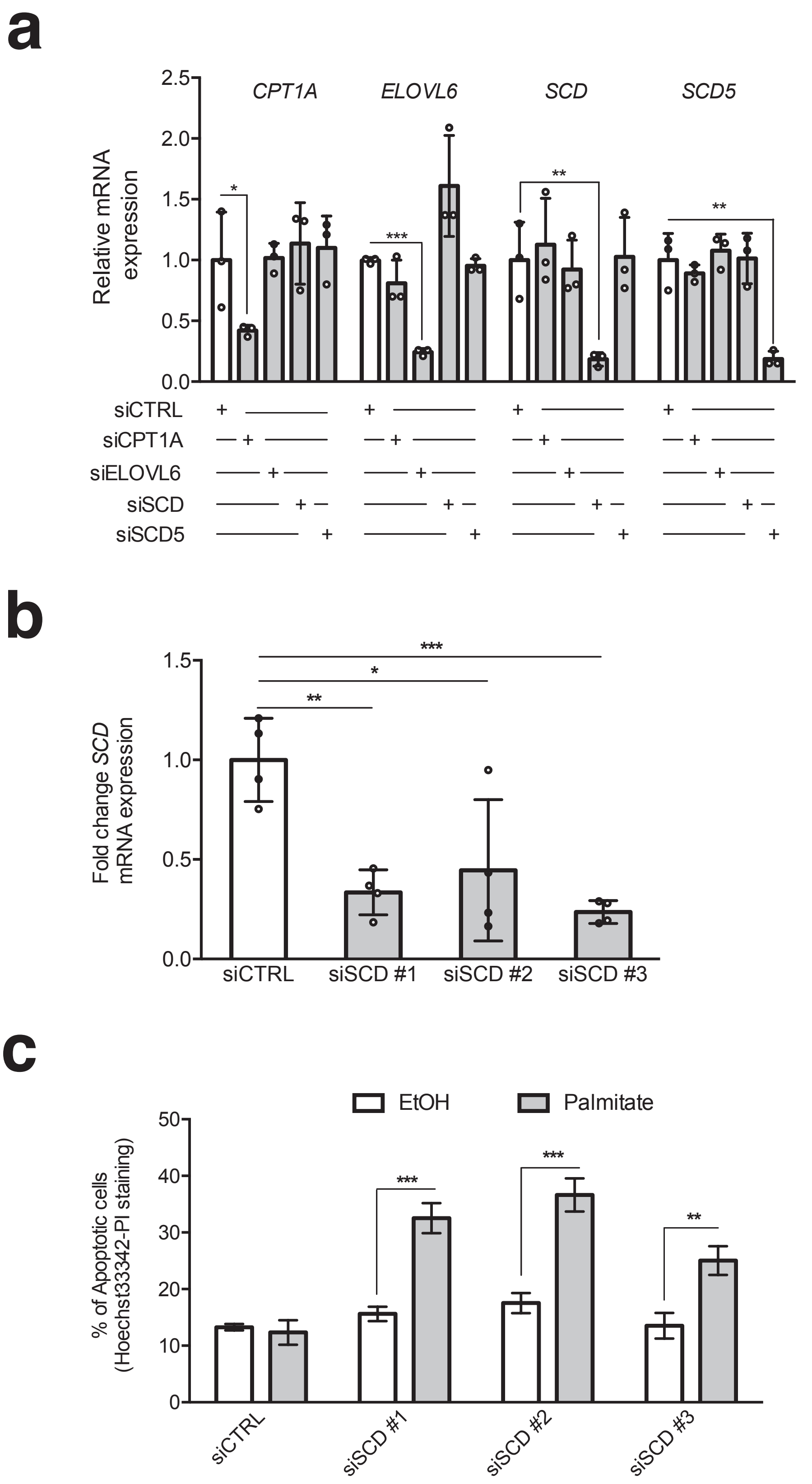

(A) EndoC-ßH1 cells were transfected with siCTRL, siCPT1A, siELOVL6, siSCD or siSCD5 for 72 h. RT-qPCR data represent relative mRNA levels of *CPT1A*, *ELOVL6*, *SCD* and *SCD5* (n=3). **(B)** EndoC-ßH1 cells were individually transfected with siCTRL or individual siSCD (#1-#3) for 48h. RT-qPCR data represent relative *SCD* mRNA levels. **(C)** EndoC-ßH1 cells transfected with individual siSCD (#1-#3) for 72 h and treated with ethanol (CTRL) or 500 µmol/L palmitate for 24 h. Graph represents measurement of apoptotic cells (percentage of cells) assessed by Hoechst 33342 and PI staining. *p<0.05, **p<0.01 and ***p<0.001 relative to control as indicated on the graph. ns: not significant.

ESM Figure 2

a

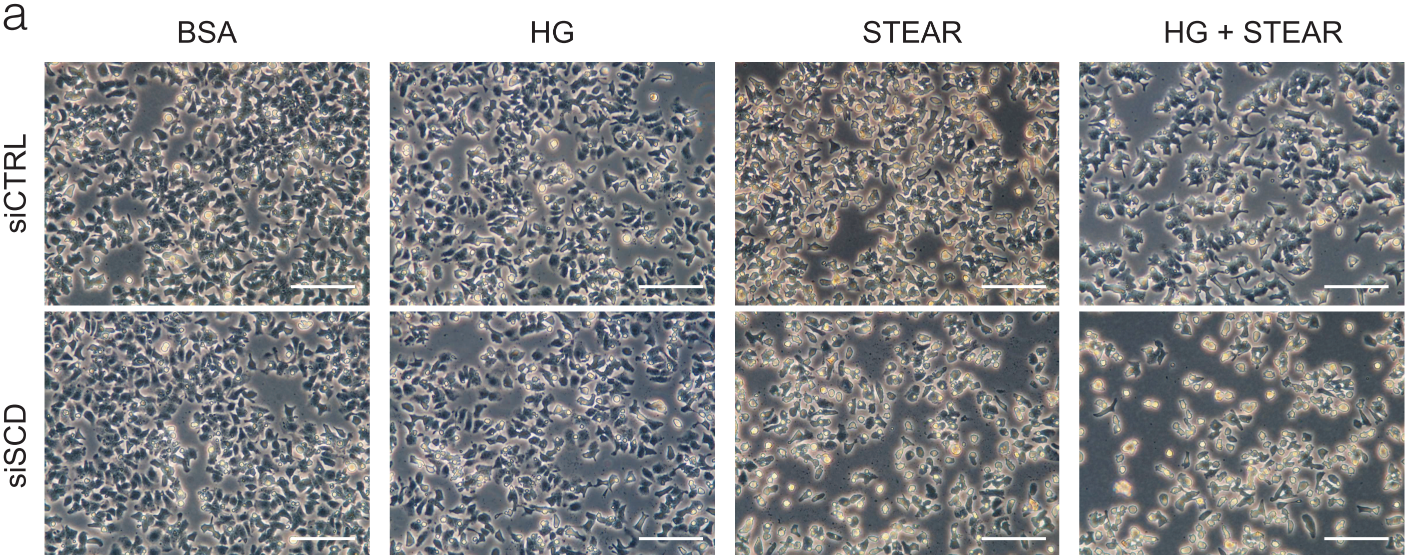

b

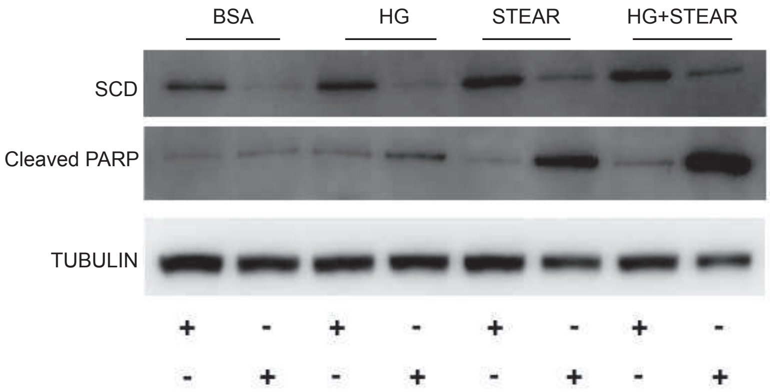

EndoC-βH1 cells were transfected with siCTRL or siSCD and treated 72 h later with BSA (control), 400 µmol/L stearate (STEAR), 30 mmol/L glucose (HG) or high glucose + stearate (HG+STEAR) for 24 h. **(A)** Representative images of EndoC-ßH1 cell morphology 24 h after treatment (scale bar 250 µm). **(B)** Western blot analysis of SCD and cleaved PARP after 24 h of treatment (n=1). *p<0.05 relative to EndoC-ßH1.

ESM Figure 3

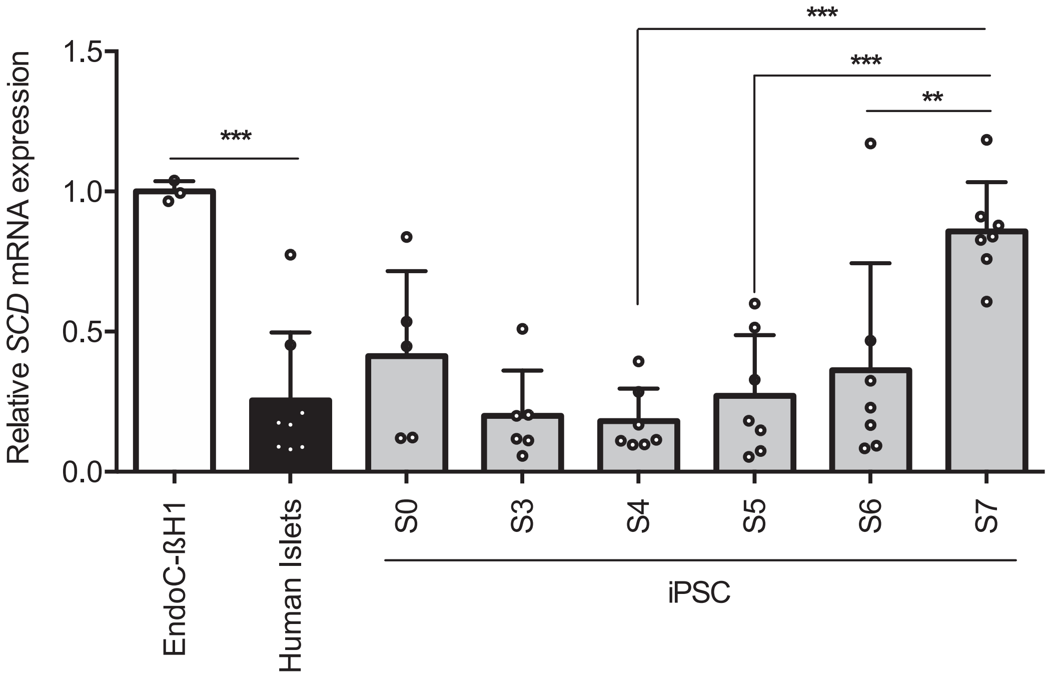

SCD mRNA expression in EndoC-ßH1 cells, human islets (n=8) and during endocrine pancreas development in the model of *in vitro* human iPSC differentiation into ß cells between stage 0 and stage 7 (n=7). S0–7, stages 0 to 7. S0: induced pluripotent stem cells, S1: OCT4$^-$/SOX17$^+$ definitive endoderm, S2: primitive gut tube, S3: posterior gut tube, S4: PDX1$^+$/NKX6.1$^+$ pancreatic progenitors, S5: endocrine progenitors, S6: endocrine cells, S7: maturing ß cells. **<p0.01 and ***p<0.001 relative to EndoC-ßH1 cells or as indicated as indicated on the graph. ns: not significant.

ESM Figure 4

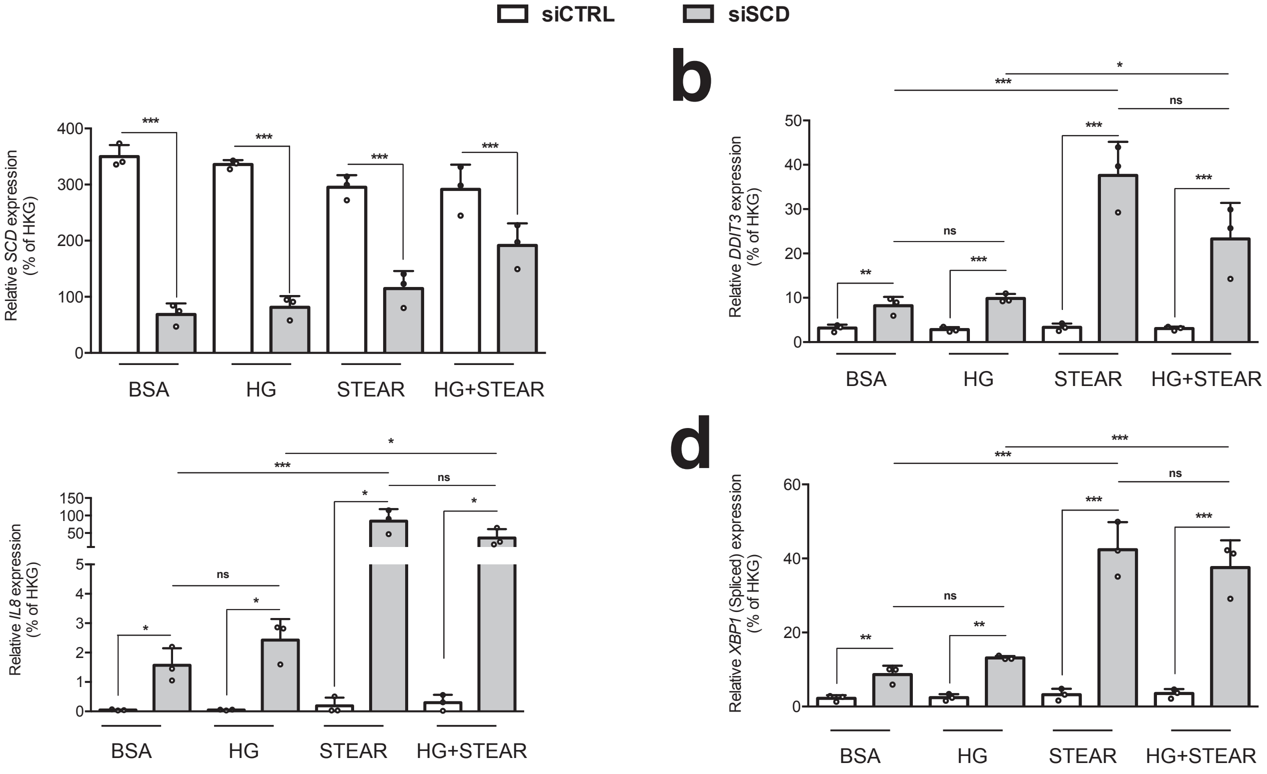

EndoC-βH1 cells were transfected with siCTRL or siSCD and treated 72 h later with BSA (control), 400 μmol/L stearate (STEAR), 30 mmol/L glucose (HG) or high glucose + stearate (HG+STEAR) for 24 h. RT-qPCR data represent relative mRNA levels of *SCD*, *IL8*, *DDIT3* and *XBP1* (spliced variant) (n=3). *p<0.05, **p<0.01 and ***p<0.001 relative to control as indicated on the graph. ns: not significant

ESM Figure 5

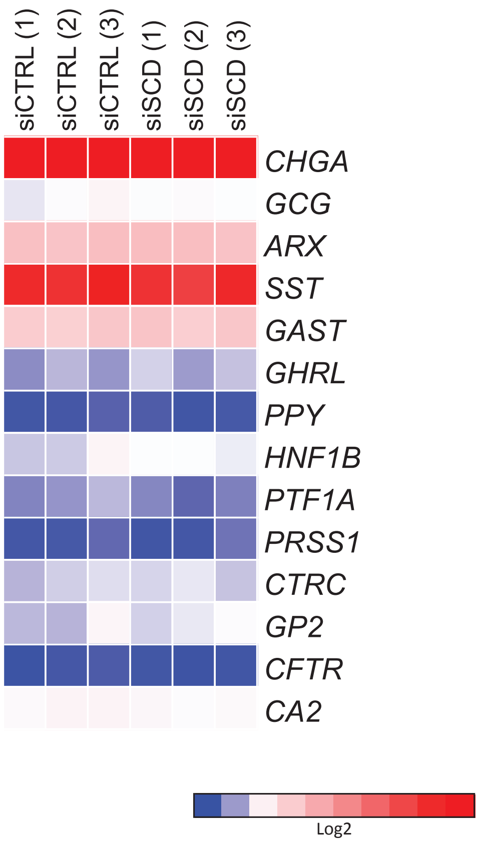

Heatmap analysis of pancreatic cell types genes upon SCD downregulation analysed by transcriptomic microarrays.

ESM checklist

Diabetologia

Checklist for reporting human islet preparations used in research

Adapted from Hart NJ, Powers AC (2018) Progress, challenges, and suggestions for using human islets to understand islet biology and human diabetes. Diabetologia https://doi.org/10.1007/s00125-018-4772-2

Islet preparation	1	2	3	4	5	6	7	8[a]
**MANDATORY INFORMATION**								
Unique identifier	21/12/15	16/12/16	27/7/17	2/8/17	13/12/17	14/2/19	28/2/19	6/4/19
Donor age (years)	76	53	64	74	71	66	60	69
Donor sex (M/F)	F	F	F	M	F	M	M	F
Donor BMI (kg/m^2)	27	NA	22	26	31	28	23	31
Donor HbA_{1c} or other measure of blood glucose control (1)	275	144	204	171	158	132	170	132
Origin/source of islets[b]	Pisa, Italy	Pisa, Italy	Pisa, Italy	Pisa, Italy	Pisa, Italy	Pisa, Italy	Pisa, Italy	Pisa, Italy
Islet isolation centre	Marchetti's Lab	Marchetti's Lab	Marchetti's Lab	Marchetti's Lab	Marchetti's Lab	Marchetti's Lab	Marchetti's Lab	Marchetti's Lab
Donor history of diabetes?	No	No	No	No	No	No	No	No
**If Yes, complete the next two lines if this information is available**								
Diabetes duration (years)								
Glucose-lowering therapy at time of death[c]								
**RECOMMENDED INFORMATION**								
Donor cause of death	Cerebral hemorrhage	Cerebral hemorrhage	Cerebral hemorrhage	Cerebral hemorrhage	Cerebral hemorrhage	Cerebral hemorrhage	Stroke	Postanoxic encephalopathy
Warm ischaemia time (h)								
Cold ischaemia time (h)	16	17	18	13	18	15	6	12
Estimated purity (%, by insulin immunostaining)	52	49	42	46	46	65	48	31
Estimated viability (%)								
Total culture time (h)[d]	48	144	168	168	192	168	168	120
Glucose-stimulated insulin secretion or other functional measurement[e] (2)	2.47	2.7	NA	3.1	2.42	To be measured	To be measured	To be measured
Handpicked to purity? Please select yes/no from drop down list								
Additional notes								

[a]If you have used more than eight islet preparations, please complete additional forms as necessary
[b]For example, IIDP, ECIT, Alberta IsletCore
[c]Please specify the therapy/therapies
[d]Time of islet culture at the isolation centre, during shipment and at the receiving laboratory
[e]Please specify the test and the results

(1) Mean glycemia during ICU stay (mg/dl)
(2) Expressed as insulin stimulation index = insulin release at 16.7 mM glucose/insulin release at 3.3 mM glucose (static iincubation)
